# Heterogeneity in Kawasaki disease patients with coronary artery abnormalities investigated by data-driven cluster analysis

**DOI:** 10.1038/s41390-025-04205-8

**Published:** 2025-06-20

**Authors:** Yuto Sunaga, Yohei Hasebe, Natsumi Kikuchi, Masashi Yoshizawa, Yosuke Kono, Nobuyuki Katsumata, Takeshi Inukai

**Affiliations:** 1 Department of Pediatrics, University of Yamanashi, Chuo, Yamanashi Japan; 2 Department of Neonatology, Yamanashi Prefectural Central Hospital, Kofu, Yamanashi Japan

## Abstract

**Background:**

To clarify the clinical characteristics associated with coronary artery abnormality (CAA) in the acute phase of Kawasaki disease (KD) cases, we performed a cluster analysis.

**Methods:**

A retrospective cohort study of 103 KD cases that developed CAA (maximum Z-score for any coronary arteries >2.5) within 30 days after disease onset was conducted among 726 consecutive KD cases. The hierarchical cluster analysis was performed based on 20 continuous variables before treatment.

**Results:**

101 out of 103 cases were clustered into 4 subgroups. Cluster 1 was a younger group characterized by leukocytosis and elevated serum triglyceride levels with frequent coronary artery dilation before treatment and good initial treatment responses. Cluster 2 was a younger group characterized by anemia and lower serum albumin and total cholesterol levels. Cluster 3 was an older group with frequent coronary artery dilation before treatment. Cluster 4 was an older group characterized by elevated serum aspartate aminotransferase, alanine aminotransferase, and total bilirubin levels, with poor treatment response and the lowest incidence of coronary artery dilatation before treatment.

**Conclusion:**

We identified four subgroups with distinct features, suggesting different backgrounds for CAA development. Consideration of possible heterogeneity might be helpful for a better understanding of the pathophysiology and better treatment strategies.

**Impact:**

- To investigate possible heterogeneity in Kawasaki disease (KD) cases that developed coronary artery abnormality (CAA) in the acute phase and to evaluate associated clinical characteristics, we performed a cluster analysis in KD cases that developed CAA in the acute phase.
- Hierarchical cluster analysis in KD cases that developed CAA within 30 days after disease onset, based on clinical data before treatment, identified four subgroups with distinct features, suggesting different backgrounds for CAA development.
- Cluster analysis might be helpful for a better understanding of the pathophysiology and better treatment strategies in KD cases at high risk for developing CAAs.

## Introduction

Kawasaki disease (KD), an acute febrile illness in infants and children, is characterized by systemic vasculitis affecting medium-sized arteries, especially coronary arteries.^1,2^ Since the development of coronary artery abnormality (CAA) is a serious complication,^2,3^ it is clinically important to control as quickly as possible any inflammation causing vasculitis. Although high-dose (2 g/kg) intravenous immunoglobulin (HD-IVIG) therapy has been established as a standard initial treatment,^2,4^ the development of CAA in the acute phase and residual CAA beyond the acute phase remains unsolved. Indeed, in a recent nationwide survey in Japan,^5^ approximately 8% and 2% of the KD patients developed CAA in the acute phase and had a residual CAA beyond the acute phase, respectively. Thus, to further improve therapeutic outcomes regarding the development of CAA, it is important to clarify the clinical characteristics of high-risk cases for developing CAA.

Recently, the utility of hierarchical clustering analysis by the machine learning approach has been demonstrated for assisting the clinical subgroups of diverse diseases.^6^ In a series of KD cases, Wang H et al. applied an unsupervised, data-driven cluster analysis and successfully identified four subgroups with distinct clinical features and treatment outcomes.^7^ In the present study, to more straightforwardly identify clinical characteristics associated with the development of CAA through a different approach from the previous report by Wang H et al.^7^ we performed a cluster analysis in 103 KD cases who developed CAA (Z-score >2.5).^8^ in the acute phase among 726 consecutive KD cases treated with an identical treatment protocol. We identified four subgroups and evaluated their association with clinical features and therapeutic outcomes.

## Materials and Methods

### Study participants

The study is a retrospective review of 726 consecutive KD cases in Japan who were diagnosed between July 2015 and December 2022 (7.5 years) in all 11 inpatient facilities for the care of pediatric patients in Yamanashi, as well as in 1 facility in Nagano (Supplementary Table 1). The study was performed with the central approval of the Research Ethics Committee of the University of Yamanashi Hospital (Approval Number 1698). The registration database was constructed with anonymized clinical records, which were provided every year from each facility under the agreement of each ethics committee. Diagnosis of KD was retrospectively confirmed based on criteria defined in the sixth edition of the Japanese Kawasaki Disease Diagnostic Guidelines.^8^ All 726 cases, including 75 incomplete KD cases, were treated within 9 days after disease onset. The first day of illness was defined as the day when at least one of the major symptoms appeared. During the COVID-19 pandemic (from March 2020 to December 2022), 169 cases were diagnosed with KD, none of whom were considered to be multisystem inflammatory syndrome in children (MIS-C) due to negative SARS-CoV-2 test (polymerase chain reaction analysis or antigen test) results on admission and no recognizable direct contact with COVID-19 cases within 2 months prior to diagnosis.

Echocardiographic evaluation was routinely performed before IVIG treatment for all the cases enrolled in this cohort. Additionally, echocardiographic evaluation was performed 48 hours after the initial treatment, before and after any additional treatment, and before discharge from the hospital. Further evaluation was performed at least every few days in cases that developed coronary artery abnormalities, until it was normalized in the acute phase. The development of coronary artery dilation was defined whenever the body surface area-adjusted *Z* score of any coronary arteries (left main, anterior descending, and circumflex arteries and right coronary arteries) was > 2.0, and the development of CAA was defined whenever it was > 2.5.^8^ When CAA was developed in the patients who were treated in the facilities other than University of Yamanashi Hospital in the acute phase (within 30 days after disease onset), they were transferred to the University of Yamanashi Hospital for further cardiac evaluation by pediatric cardiologists and acute-phase treatment including plasma exchange if required. Then, they were followed up in the outpatient clinic of the University of Yamanashi Hospital. Regular cardiac echo evaluation of the case with CAA was independently performed by at least two pediatric cardiologists.

### Treatment of Kawasaki disease

All facilities performed an identical treatment protocol, as confirmed in the annual meeting, in which a representative pediatrician from each facility participated. All patients were treated with 2 g/kg/dose of IVIG in combination with oral aspirin (30 mg/kg/day) or its analogs as a first-line treatment immediately after the definitive diagnosis based on the criteria.^8^ IVIG treatment was completed within 24 hours after diagnosis, without any other anti-inflammatory agents, including steroids and cyclosporine A. The response to the initial treatment was evaluated 48 hours after the initiation of IVIG administration. The cases were considered to be ‘IVIG resistant’ when their body temperature was over 37.5 °C and serum C-reactive protein (CRP) level was higher than half of the peak value.^9,10^

The second-line treatment was mainly intravenous administration of infliximab at 5 mg/kg. Meanwhile, additional IVIG was administered as the second-line treatment in cases aged under 12 months old, in cases complicated with any active infection, or in cases with disease that developed within 12 weeks after live virus vaccination. The patients were considered to be resistant to the second-line treatment when the fever was unresolved within 48 hours after initiation of the second-line treatment. All cases resistant to the second-line treatment were transferred to the University of Yamanashi Hospital, and plasma exchange was performed in cases refractory to further IVIG treatment as the third-line treatment or in cases with rapidly progressive CAA.^11^

### Machine learning

Cluster analysis using the Python software (version 3.11.5) was performed in 103 cases that developed CAA in the acute phase. For any missing laboratory data, the median value was complementary used in the machine learning. In the first step, using 33 continuous variables before starting initial treatment (Supplementary Table 2), we evaluated Spearman’s rank correlation coefficient between each pair of the 33 continuous variables. When the r-value between two variables was 0.5 or higher, we chose the variable with fewer missing values. Resultantly, we identified 20 variables (Table 1 and Supplementary Fig. 1). The number of missing values for each of the 20 items is indicated in Supplementary Table 3. In the second step, we normalized input data by a standard scaler.^12,13^, and performed a principal component analysis to ensure that the contribution ratio remained above 80%.^14–16^ In the final step, we performed hierarchical cluster analysis.^16,17^ The Hopkins statistic was used to assess the clustering tendency of data.^18^

**Table 1:** List of the variables used for clustering analysis.

Categories	Variables
Demographic features	Age, days of initial treatment (start day), The number of major KD symptoms (symptoms)
Laboratory data	White blood cell count (WBC); platelet count (Plt); serum levels of C-reactive protein (CRP), albumin (Alb), sodium, potassium, aspartate aminotransferase (AST), total bilirubin, blood urea nitrogen (BUN), creatine kinase (CK), total cholesterol (T. cholesterol), high density lipoprotein cholesterol (HDL chol), and triglyceride (TG); PT-INR, fibrinogen, D-dimer value
Echocardiographic parameters	Maximum coronary artery Z-score before initial treatment (Pre-max CA Z-score)

### Statistical analysis

In comparisons of continuous variables between the two subgroups, the Mann–Whitney U test was performed when the Kruskal–Wallis rank sum test results were significant among the four subgroups. Pearson’s Chi-squared test was performed for comparisons of categorical variables. The Bonferroni method was used to compare significance levels for multiple comparisons. All statistical analyses were performed using EZR software (version 1.41; Saitama Medical Center, Jichi Medical University, Saitama, Japan).^19^

## Results

During the study period, among 726 consecutive cases, 103 cases (14.2%) developed CAA (Z-score > 2.5)^8^ within 30 days after disease onset; 18 cases and 3 cases had residual CAA on day 30 and 1 year after disease onset, respectively. No cases developed giant aneurysm during the study period. When the demographic characteristics and laboratory findings were analyzed (Supplementary Table 4), the 103 cases with CAA were significantly younger; had significantly higher rates of incomplete KD and treatment resistance; higher maximum coronary artery Z-scores before initial treatment (Pre-max CA Z-score); lower levels of hemoglobin, total cholesterol, and high-density lipoprotein (HDL) cholesterol; and higher levels of serum albumin, alanine aminotransferase (ALT), and CRP than the 623 cases without CAA.

**Fig. 1:**
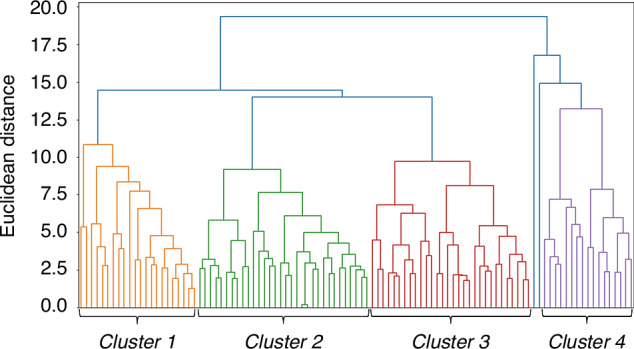
Dendrogram of the hierarchical map in the hierarchical clustering analysis. The horizontal axis indicates each case in each subgroup, and the vertical axis indicates Euclidean distance.

In the cluster analysis of the 103 cases with CAA, we identified four subgroups (Clusters 1 to 4) (Fig. 1 and Supplementary Fig. 2) with a reliable cluster structure (Hopkins statistic: 0.76) except for two cases; one developed KD shock syndrome and macrophage activation syndrome, and the other developed severe hepatic dysfunction. Thus, we focused on the 101 cases with CAA that were clustered into Cluster 1 (22 cases, 21.8%), Cluster 2 (32 cases, 31.7%), Cluster 3 (30 cases, 29.7%), and Cluster 4 (17 cases, 16.8%). In each facility (Supplementary Table 1), there were some variations in the distribution of each cluster, but no statistically significant differences were observed as a whole (*p* = 0.23 in Chi-square test).

**Fig. 2:**
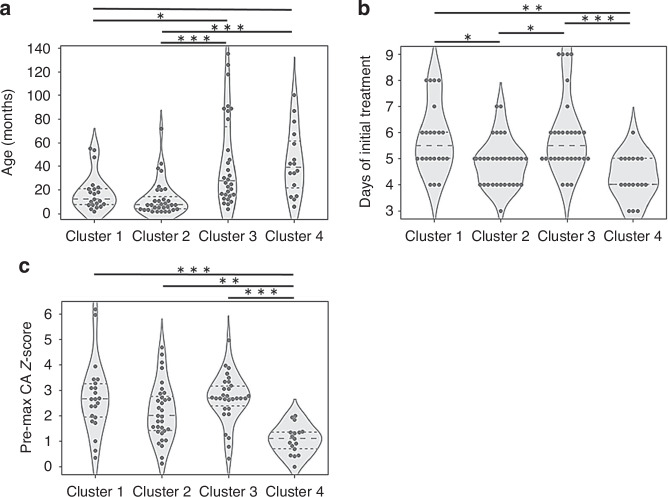
Violin diagrams with swarm plots in four subgroups. **a** age, **b** days of initial treatment, and **c** max coronary artery Z-score before treatment (Pre-max CA Z-score). *p < 0.05, **p < 0.01, ***p < 0.001 in the Mann–Whitney U test.

In the demographics at diagnosis (Table 2), the cases in Clusters 1 and 2 were significantly younger than those in Clusters 3 and 4 (Fig. 2a). Days of initial treatment were significantly earlier in Cluster 4 than in the other subgroups (Fig. 2b). There were no significant differences in the rate of incomplete KD among the four subgroups.

**Table 2:** Characteristics of each subgroup.

Characteristics	Total (*n* = 101)	Cluster 1 (*n* = 22)	Cluster 2 (*n* = 32)	Cluster 3 (*n* = 30)	Cluster 4 (*n* = 17)	*p* value*
Age; months	17 (7–38)	12 (7–21)	7 (4–14)	28 (16–74)	39 (22–61)	<0.01
<6 months; *n* (%)	17 (16.8)	3 (13.6)	13 (40.6)	1 (3.3)	0 (0.0)	<0.01
<12 months; *n* (%)	38 (37.6)	10 (45.5)	23 (71.8)	4 (13.3)	1 (5.9)	<0.01
Male; *n* (%)	53 (52.4)	12 (54.5)	18 (56.2)	15 (50.0)	8 (47.0)	0.13
Days of initial treatment; day	5 (4–6)	5 (5-6)	5 (4-5)	5.5 (5-6)	4 (4-5)	<0.01
Incomplete KD; *n* (%)	19 (18.8)	4 (18.2)	9 (28.1)	4 (13.3)	2 (11.8)	0.40
**Treatment response**						
IVIG resistance; *n* (%)	30 (29.7)	2 (9.1)	8 (25.0)	8 (26.7)	12 (70.6)	<0.01
Resistance of 2nd line treatment; *n* (%)	20 (19.8)	0 (0.0)	7 (21.8)	3 (10.0)	10 (58.8)	<0.01
Plasma exchange; *n* (%)	12 (11.9)	0 (0.0)	6 (18.8)	1 (3.3)	5 (29.4)	<0.01
**Coronary Artery (CA) data at diagnosis**						
Pre-max CA diameter; mm	2.4 (2.0–2.7)	2.3 (2.0–2.6)	2.1 (1.9–2.5)	2.6 (2.4–3.1)	2.2 (1.9–2.4)	<0.01
Pre-max CA Z-score	2.31 (1.33-2.89)	2.65 (1.94–3.24)	1.99 (1.40–2.76)	2.69 (2.37–3.16)	1.08 (0.69–1.35)	<0.01
Pre-max CA Z-score >2.0; *n* (%)	58 (57.4)	15 (68.1)	16 (50.0)	26 (81.3)	1 (5.9)	<0.01
Pre-max CA Z-score >2.5; *n* (%)	47 (46.5)	12 (54.5)	13 (40.6)	22 (68.8)	0 (0.0)	<0.01
Pre-max CA Z-score >5.0; *n* (%)	2 (2.0)	2 (9.1)	0 (0.0)	0 (0.0)	0 (0.0)	0.07
**CA data in acute phase**						
Max CA Z-score within 30 days	3.07 (2.69–3.97)	3.37 (2.74–4.04)	2.92 (2.68–4.11)	2.88 (2.68–3.38)	3.10 (2.72–4.10)	0.46
Max CA Z-score >5.0 (within 30 days); *n* (%)	13 (12.9)	3 (13.6)	5 (15.6)	1 (3.3)	4 (23.5)	0.22
**CA data after 30 days**						
CAA after 30 days; *n* (%)	16 (15.8)	5 (22.7)	6 (18.8)	3 (10.0)	2 (11.8)	0.58
CAA after 6 months; *n* (%)	8 (7.9)	1 (4.5)	5 (15.6)	0 (0.0)	2 (11.8)	0.12
CAA after 1 year; *n* (%)	3 (3.0)	1 (4.5)	1 (3.1)	0 (0.0)	1 (5.9)	0.66
**Blood Laboratory finding**						
White blood cell count (WBC); ×10^3^ /µL	13.9 (10.6–16.4)	17.3 (15.4–19.8)	12.7 (10.0–14.8)	12.2 (10.5–14.0)	14.6 (9.5–15.7)	<0.01
Hemoglobin; g/dL	11.2 (10.3–11.9)	11.0 (10.0-11.7)	10.5 (9.3-11.2)	11.9 (11.1-12.1)	11.5 (10.5-11.8)	<0.01
Platelet count; ×10^4^ /µL	35 (27.8–40.9)	39.5 (37.0–50.8)	38.6 (30.1–44.8)	32.9 (28.1–35.3)	25.6 (23.9–27.9)	<0.01
Aspartate aminotransferase (AST); U/L	34 (27–69)	34 (30–62)	32 (21–57)	33 (27–42)	139 (37–485)	0.04
Alanine aminotransferase (ALT); U/L	30 (16–108)	32 (20–131)	26 (15–47)	20 (14–101)	136 (81–280)	<0.01
Total bilirubin; mg/dL	0.6 (0.5–0.9)	0.5 (0.5–1.0)	0.6 (0.4–0.8)	0.5 (0.5–0.7)	2.5 (0.9–3.5)	<0.01
Sodium; mmol/L	134 (132–137)	134 (132–136)	135 (133–137)	135 (133–138)	131 (129–132)	<0.01
C-reactive protein (CRP); mg/L	9.4 (5.2–13.4)	9.8 (6.6–14.5)	9.9 (5.4–12.3)	7.6 (4.0–12.6)	9.7 (9.6–16.7)	0.09
Albumin; g/dL	3.3 (3.0–3.6)	3.4 (3.1–3.5)	3.2 (2.9–3.3)	3.6 (3.4–3.8)	3.2 (2.8–3.7)	<0.01
Triglyceride; mg/dL	93 (77–123)	133 (119–156)	85 (75.5–110)	80 (72–93)	92 (64–125)	<0.01
Total cholesterol; mg/dL	134 (118–159)	158 (140–171)	121 (103–133)	142 (125–157)	124 (116–142)	<0.01
HDL cholesterol; mg/dL	30 (23–37)	26 (21–28)	27 (22–33.5)	34 (29–45)	35 (23–52)	<0.01
Gunma score	3 (1–6)	3 (2–4)	3 (2–4)	2 (0–4)	8 (6–9)	<0.01
Kurume score	2 (1–3)	1 (1–3)	2 (1–3)	1 (0–3)	4 (3–5)	<0.01
Osaka score	1 (0–2)	1 (0–2)	1 (0–1)	1 (0–1)	2 (2–2)	<0.01

Data are expressed as the median (IQR) or n (%). CAA defined maximum coronary artery Z-score >2.5. * Kruskal-Wallis or Chi-squared test.

**Fig. 3:**
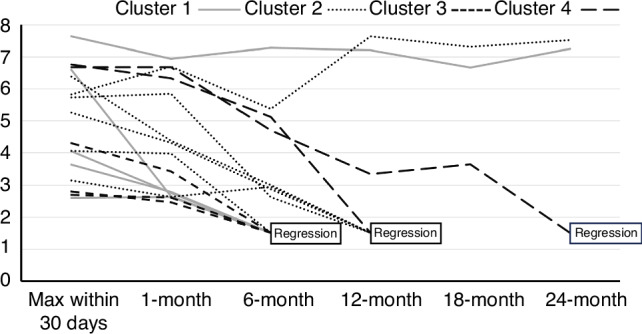
Regression of residual CAA. The horizontal axis indicates the time from onset, and the vertical axis indicates Z-score. CAA was considered to be regressed when the Z-score fell below 2.5.

In the treatment responses (Table 2), IVIG resistance in the first and second-line treatments was significantly more common in Cluster 4 but uncommon in Cluster 1. Plasma exchange was significantly more common in Clusters 2 and 4 than in Clusters 1 and 3. In the cardiac echo evaluation (Table 2), maximum coronary artery Z-scores before initial treatment (Pre-max CA Z-scores) were significantly lower in Cluster 4 than in the other subgroups (Fig. 2c). Although it was the highest in Cluster 1, no statistically significant differences were observed in the incidence of residual CAA 30 days from disease onset among the four subgroups. In echocardiographic follow-up of residual CAA (Table 2 and Fig. 3), regression within six months from disease onset was frequently observed in Clusters 1 and 3, while it was rarely observed in Clusters 2 and 4. Regression of residual CAA within six months after diagnosis was observed in seven of eight cases with max Z-score within day 30 < 5.0, while it was observed in only one of eight cases with max Z-score within day 30 > 5.0.

**Fig. 4:**
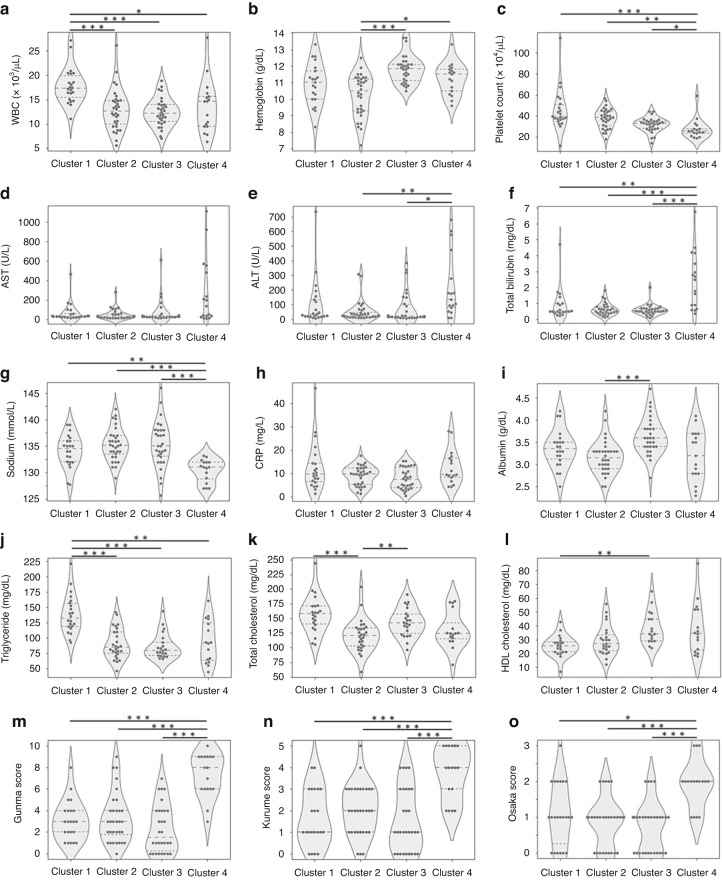
Violin diagrams with swarm plots of 11 blood test items and 3 IVIG resistance prediction scores in four subgroups. **a** white blood cell count (WBC), **b** hemoglobin, **c** platelet count, **d** aspartate aminotransferase (AST), **e** alanine aminotransferase (ALT), **f** total bilirubin, **g** sodium, **h** C-reactive protein (CRP), **i** albumin, **j** triglyceride, **k** total cholesterol, **l** high density lipoprotein (HDL) cholesterol, **m** Gunma score, **n** Kurume score and **o** Osaka score. *p < 0.05, **p < 0.01, ***p < 0.001 in the Mann–Whitney U test.

In the peripheral blood counts (Table 2), leukocytosis was the most remarkable in Cluster 1 among the four subgroups (Fig. 4a), while anemia was the most remarkable in Cluster 2 (Fig. 4b). Thrombocytosis was significantly less remarkable in Cluster 4 than in the other subgroups (Fig. 4c). Regarding blood chemistry (Table 2), levels of serum aspartate aminotransferase (AST), ALT, and total bilirubin were significantly higher in Cluster 4 than in the other subgroups (Fig. 4d–f), while levels of serum sodium were significantly lower in Cluster 4 (Fig. 4g). There were no significant differences in the level of serum CRP among the four subgroups (Fig. 4h). Serum albumin level was relatively lower in Cluster 2 (Fig. 4i), and a statistically significant difference was observed between Clusters 2 and 3. As for the levels of serum lipid, triglyceride (TG) was significantly higher in Cluster 1 than in the other subgroups (Fig. 4j), total cholesterol was significantly lower in Cluster 2 than in Clusters 1 and 3 (Fig. 4k), and HDL cholesterol was relatively higher in Clusters 3 and 4 than in Clusters 1 and 2 (Fig. 4l). In the scoring systems for predicting IVIG resistance (Table 2), all three scores in Gunma,^20^ Kurume,^9^ and Osaka.^21^ systems were significantly higher in Cluster 4 than in the other subgroups (Fig. 4m–o).

In summary, Cluster 1 was characterized by leukocytosis and elevated serum TG levels in a relatively younger-aged group with a higher Z-score of coronary artery dilation before treatment and a relatively good response to initial IVIG treatment. Cluster 2 was characterized by anemia and lower serum total cholesterol level in a relatively younger-aged group with an average response to initial IVIG treatment. Cluster 3 was a relatively older-aged group lacking characteristic laboratory findings, with the highest Z-score of coronary artery dilation before treatment, but earlier regression of residual CAA. Cluster 4 was characterized by elevated levels of serum AST, ALT, and total bilirubin and decreased levels of sodium in a relatively older-aged group with poor response to initial IVIG treatment, despite the lowest Z-score of maximum coronary artery dilation before treatment.

## Discussion

In this study, we performed clustering analysis in 103 KD cases who developed CAA within 30 days after disease onset, and identified four subgroups with different clinical characteristics and treatment responses. In the present study, we included serum lipid levels in the clustering analysis, since serum lipid levels have been identified as one of the predicting factors for IVIG-resistance in several previous studies, including ours.^22–24^ Dyslipidemia, including higher levels of TG and lower levels of total cholesterol, is associated with the severity of systemic inflammation in KD, although the underlying mechanism remains unclear. Indeed, each cluster showed a characteristic pattern in serum lipid levels. However, we could not confirm a consistent association of dyslipidemia with the development and regression of CAA as well as IVIG-resistance in each cluster, suggesting that further evaluation in a larger cohort is necessary for this issue.

Among the four subgroups, Cluster 4 was the most characteristic subgroup. Although all the cases in this cluster analysis developed CAA within 30 days after disease onset, coronary artery Z-scores before treatment were significantly lower in Cluster 4 compared to the other three subgroups. In other words, the cases in Cluster 4 most frequently developed CAA during treatment. Indeed, among the four subgroups, resistance to the first and second-line treatments was most frequent in Cluster 4, and, subsequently, plasma exchange was performed more frequently in Cluster 4 than in the other three subgroups. However, in Cluster 4, maximum coronary artery Z- scores within 30 days after disease onset were fairly similar to those in the other three groups, and the incidence of residual CAA on day 30 after disease onset was relatively lower among the four subgroups. Our observations suggested that, in Cluster 4, the second-line treatment and plasma exchange could be effective in preventing further progression of CAA, although we were unable to directly clarify the underlying mechanisms for this association. Cluster 4 was characterized by elevated levels of serum AST, ALT, and total bilirubin and decreased levels of serum sodium. Cluster 4 also showed significantly higher scores in the scoring systems for IVIG resistance prediction, despite the lower incidence of CAA at diagnosis. Thus, it is feasible to predict that the currently ongoing intensified initial IVIG treatment in combination with other anti-inflammatory agents.^25–29^ might be effective in preventing CAA development in cases characterized by clinical features observed in Cluster 4. In contrast to Cluster 4, Cluster 3 seemed to be the most severe in terms of pre-treatment coronary artery dilatation. However, regression of residual CAA was observed within six months in all three cases with residual CAA, suggesting that further intensification of initial treatment might not be required in this cluster.

Recently, accumulated evidence has shown that a higher Z-score from any coronary artery before initial treatment is associated with a higher risk of developing CAA.^30–36^ Indeed, in our consecutive 726 KD cases, the 103 cases with CAA in the acute phase showed significantly higher Z-scores of any coronary arteries before initial treatment than the 623 cases without it. Among the 101 cases with CAA clustered into 4 subgroups, 58 cases (57.4%) showed coronary artery dilation before treatment (Z-score was higher than 2.0). Of note, the majority of these cases with dilated coronary arteries before treatment (57/58 cases) were clustered into Clusters 1–3, and the incidence of residual CAA on day 30 after disease onset was relatively higher in Clusters 1 and 2, despite their younger age onset and lower scores in the scoring systems. In terms of regression of residual CAA, regression within six months from disease onset was common in Clusters 1 and 3, while it was uncommon in Clusters 2 and 4. Thus, when coronary artery dilation was confirmed before treatment in the low-risk cases for IVIG resistance, such as Clusters 1 and 2, intensification of initial treatment by a combination of other anti-inflammatory agents might be more crucial in Cluster 2, rather than in Cluster 1, to prevent CAA development. However, even in Cluster 1, one in five cases with residual CAA had persistent CAA over two years after diagnosis, suggesting a requirement for other biomarker(s) for the prediction of persistent residual CAA in Cluster 1.

Clustering analysis is a method of unsupervised machine learning, and its utility in clinical medicine has been increasingly confirmed.^37–40^ In the recent clustering analysis of a large series of KD cases by Wang H et al.^7^ four subgroups were identified: (1) liver subgroup characterized by hepatobiliary involvement with elevated levels of ALT; gamma-glutamyl transferase, and total bilirubin showing the lowest rate of coronary artery aneurysm and the highest rate of IVIG resistance; (2) band subgroup characterized by the highest band neutrophil count showing a high KD shock rate; (3) node subgroup characterized by cervical lymphadenopathy with higher markers of inflammation and the lowest age-adjusted hemoglobin Z-scores; and (4) young subgroup characterized by young aged onset showing the highest rate of coronary artery aneurysm but the lowest rate of intravenous immunoglobulin resistance. Among these four subgroups identified by Wang H et al.^7^ although not directly comparable to our observations due to different patient population and treatment, the characteristics in the liver subgroup were similar to those in Cluster 4 in the present study, suggesting the importance and prevalence of this clinical entity as a treatment-resistant subgroup in older children. These subgroups of the cases were commonly characterized by elevated serum ALT and total bilirubin levels in the older-age cases with the highest rate of IVIG resistance, suggesting the importance of these characteristics and laboratory findings in the clinical management of KD cases.

This study has several limitations. First, the majority of the cases in the present study were of Japanese ethnicity, and it was relatively small sample size. Second, although the efficacy of intensive initial IVIG treatment combined with other anti-inflammatory agents has been recently reported for high-risk KD patients,^25–29^ first-line treatment in the present study was a standardized IVIG protocol in combination with oral aspirin or its analogs. Third, this study was retrospectively performed based on the registration database of clinical records. Fourth, several biomarkers for IVIG-resistance, such as eosinophil count.^41,42^ were not included in this study, suggesting that such missing factors might somehow affect the pattern of clustering. Finally, the long-term outcome of CAA was not fully evaluated.

In conclusion, our cluster analysis of the KD cases that developed CAA in the acute　phase identified four subgroups with distinct clinical characteristics and treatment responses, suggesting different backgrounds for disease progression in a single disease entity. In particular, common characteristics between our Cluster 4 and ‘liver subgroup’ identified by Wang H et al.^8^ indicate the importance and prevalence of this clinical entity. Clinically, our observations also suggest that intensification of initial treatment with additional anti-inflammatory agents might be more crucial in Clusters 2 and 4 rather than in Clusters 1 and 3. Although further validation is needed, consideration of possible heterogeneity in the progression of CAA may be helpful for a better understanding of pathophysiology and, subsequently, better treatment strategies to protect KD cases from CAA development.

## Supplementary information

Supplementary Fig. S1

Supplementary Fig. S2

Supplementary Table. S1

Supplementary Table. S2

Supplementary Table. S3

Supplementary Table. S4

## Data Availability

The datasets generated during and/or analyzed during the current study are not publicly available due to the risk of revealing the identity of the subjects, but are available from the corresponding author on reasonable request.
