# Supplementary material for: Heterogeneity in Kawasaki disease patients with coronary artery abnormalities investigated by data-driven cluster analysis
Source: Pediatr Res. 2025 Jun 20;98(5):1809–16. doi: 10.1038/s41390-025-04205-8 (PMC12602351; doi:10.1038/s41390-025-04205-8)
Supplement: Supplementary file 1 — Supplementary Fig. S1 [file 41390_2025_4205_MOESM1_ESM.pdf]

	Age	Height	Weight	BSA	Start day	Symptoms	WBC	Hb	Plt	CRP	TP	Alb	CK	AST	ALT	LDH	Total bilirubin	BUN	Cre	Sodium	Potassium	Chloride	T. choleste-rol	HDL choleste-rol	Triglyceri-de	LDL choleste-rol	Non HDL choleste-rol	IgG	PT-INR	Fibrinogen	D-dimer	Pre-max CA diameter	Pre-max CA Z-score
Age	1.00																																
Height	0.95	1.00																															
Weight	0.94	0.97	1.00																														
BSA	0.95	0.99	0.99	1.00																													
Start day	0.24	0.24	0.27	0.26	1.00																												
Symptoms	0.19	0.15	0.13	0.13	-0.18	1.00																											
WBC	-0.13	-0.16	-0.15	-0.15	-0.09	0.10	1.00																										
Hb	0.61	0.59	0.60	0.60	0.20	0.25	-0.06	1.00																									
Plt	-0.46	-0.46	-0.46	-0.46	-0.02	-0.19	0.39	-0.39	1.00																								
CRP	0.08	0.09	0.12	0.10	0.01	-0.06	0.03	-0.08	-0.13	1.00																							
TP	0.53	0.49	0.46	0.49	0.16	0.22	0.19	0.46	-0.08	-0.03	1.00																						
Alb	0.18	0.11	0.10	0.12	-0.07	0.28	0.13	0.34	0.00	-0.26	0.71	1.00																					
CK	0.02	0.03	0.02	0.03	0.09	0.03	-0.13	0.08	0.00	-0.01	0.31	0.27	1.00																				
AST	0.06	0.01	0.00	0.01	-0.28	0.15	0.04	0.02	-0.15	0.00	0.00	0.19	0.20	1.00																			
ALT	0.05	0.00	0.02	0.02	-0.29	0.15	0.08	0.01	-0.16	0.09	-0.11	0.05	-0.02	0.80	1.00																		
LDH	-0.03	-0.12	-0.09	-0.10	-0.22	0.20	0.08	0.18	-0.06	-0.20	0.17	0.38	0.30	0.55	0.29	1.00																	
Total bilirubin	0.15	0.14	0.13	0.14	-0.39	0.26	0.09	-0.05	-0.18	0.12	-0.01	0.10	-0.04	0.37	0.47	0.17	1.00																
BUN	0.36	0.33	0.32	0.33	-0.07	0.16	0.04	0.36	-0.20	0.13	0.48	0.44	0.18	0.18	0.13	0.20	0.21	1.00															
Cre	0.67	0.69	0.70	0.70	0.07	0.04	-0.16	0.38	-0.33	0.17	0.32	0.05	0.19	0.12	0.16	-0.10	0.21	0.40	1.00														
Sodium	-0.16	-0.18	-0.20	-0.18	0.19	-0.23	-0.12	-0.10	0.30	-0.27	-0.02	0.10	0.09	-0.21	-0.24	0.03	-0.30	-0.17	-0.15	1.00													
Potassium	-0.43	-0.42	-0.39	-0.40	-0.02	-0.12	0.24	-0.09	0.34	-0.12	0.01	0.13	-0.04	-0.08	-0.13	0.12	-0.26	0.04	-0.40	0.22	1.00												
Chloride	-0.36	-0.38	-0.39	-0.38	-0.09	-0.13	-0.12	-0.21	0.16	-0.31	-0.20	0.03	-0.04	-0.08	-0.08	0.06	-0.22	-0.22	-0.27	0.65	0.19	1.00											
T. cholesterol	0.05	-0.03	-0.06	-0.04	0.15	0.02	0.08	0.04	0.15	-0.23	0.32	0.38	0.21	0.03	-0.04	0.22	0.02	0.17	-0.04	0.18	0.19	0.08	1.00										
HDL cholesterol	0.08	0.05	0.01	0.03	-0.33	0.14	-0.07	0.08	-0.09	-0.13	0.32	0.32	0.09	0.03	-0.06	0.10	0.11	0.20	0.07	-0.13	0.06	0.09	0.19	1.00									
Triglyceride	-0.11	-0.16	-0.13	-0.15	0.28	-0.04	0.20	-0.06	0.14	0.01	-0.18	-0.08	-0.01	0.11	0.14	0.06	-0.02	0.04	-0.08	-0.03	0.07	-0.06	0.30	-0.49	1.00								
LDL cholesterol	-0.04	-0.08	-0.06	-0.07	0.29	-0.06	0.08	0.01	0.24	-0.19	0.25	0.31	0.30	-0.07	-0.11	0.15	-0.04	0.14	-0.04	0.29	0.23	0.06	0.79	-0.08	0.31	1.00							
Non HDL cholesterol	-0.07	-0.14	-0.11	-0.12	0.29	-0.04	0.07	-0.01	0.23	-0.16	0.14	0.21	0.27	-0.03	-0.06	0.15	-0.01	0.12	-0.05	0.24	0.22	0.01	0.77	-0.19	0.51	0.93	1.00						
IgG	0.55	0.54	0.53	0.54	0.11	0.25	0.14	0.37	-0.25	0.06	0.47	0.23	0.14	0.10	0.13	0.17	0.25	0.27	0.37	-0.06	-0.19	-0.22	0.05	-0.05	0.02	0.03	0.04	1.00					
PT-INR	0.03	0.03	0.01	0.02	-0.31	-0.02	-0.10	-0.14	-0.08	0.04	-0.27	-0.23	-0.03	0.21	0.18	0.01	0.34	-0.08	0.14	-0.14	-0.22	0.01	-0.38	0.05	-0.17	-0.41	-0.38	-0.01	1.00				
Fibrinogen	0.22	0.26	0.26	0.26	0.00	0.01	0.13	0.15	0.04	0.27	0.22	0.05	-0.02	0.01	0.08	-0.19	0.13	0.15	0.16	-0.08	0.03	-0.19	0.01	0.08	-0.01	-0.02	-0.02	0.01	-0.05	1.00			
D-dimer	0.08	0.09	0.09	0.08	0.00	0.02	-0.02	-0.08	-0.18	0.36	-0.22	-0.34	-0.05	0.17	0.14	-0.03	0.23	-0.13	0.12	-0.31	-0.20	-0.36	-0.28	-0.18	0.11	-0.30	-0.20	0.01	0.25	-0.02	1.00		
Pre-max CA diameter	0.59	0.63	0.63	0.63	0.37	-0.10	-0.19	0.36	-0.19	0.19	0.27	-0.01	-0.05	-0.06	-0.07	-0.22	-0.03	0.16	0.38	0.04	-0.14	-0.20	-0.02	0.06	-0.14	0.01	-0.07	0.22	-0.15	0.26	0.02	1.00	
Pre-max CA Z-score	-0.01	0.00	-0.01	0.00	0.31	-0.18	-0.06	0.09	0.10	0.07	-0.02	-0.04	-0.03	-0.05	-0.10	-0.13	-0.14	-0.04	-0.12	0.22	0.16	0.03	0.04	-0.03	0.03	0.13	0.09	-0.10	-0.19	0.06	-0.04	0.68	1.00

Supplemental Fig. S1 Correlated variables of all 33 continuous variables before initial treatment. Variables with gray scale means the variable which was removed due to highly correlated pairs with the other. (Spearman’s r>0.5).
