# Supplementary material for: Heterogeneity in Kawasaki disease patients with coronary artery abnormalities investigated by data-driven cluster analysis
Source: Pediatr Res. 2025 Jun 20;98(5):1809–16. doi: 10.1038/s41390-025-04205-8 (PMC12602351; doi:10.1038/s41390-025-04205-8)
Supplement: Supplementary file 2 — Supplementary Fig. S2 [file 41390_2025_4205_MOESM2_ESM.pdf]

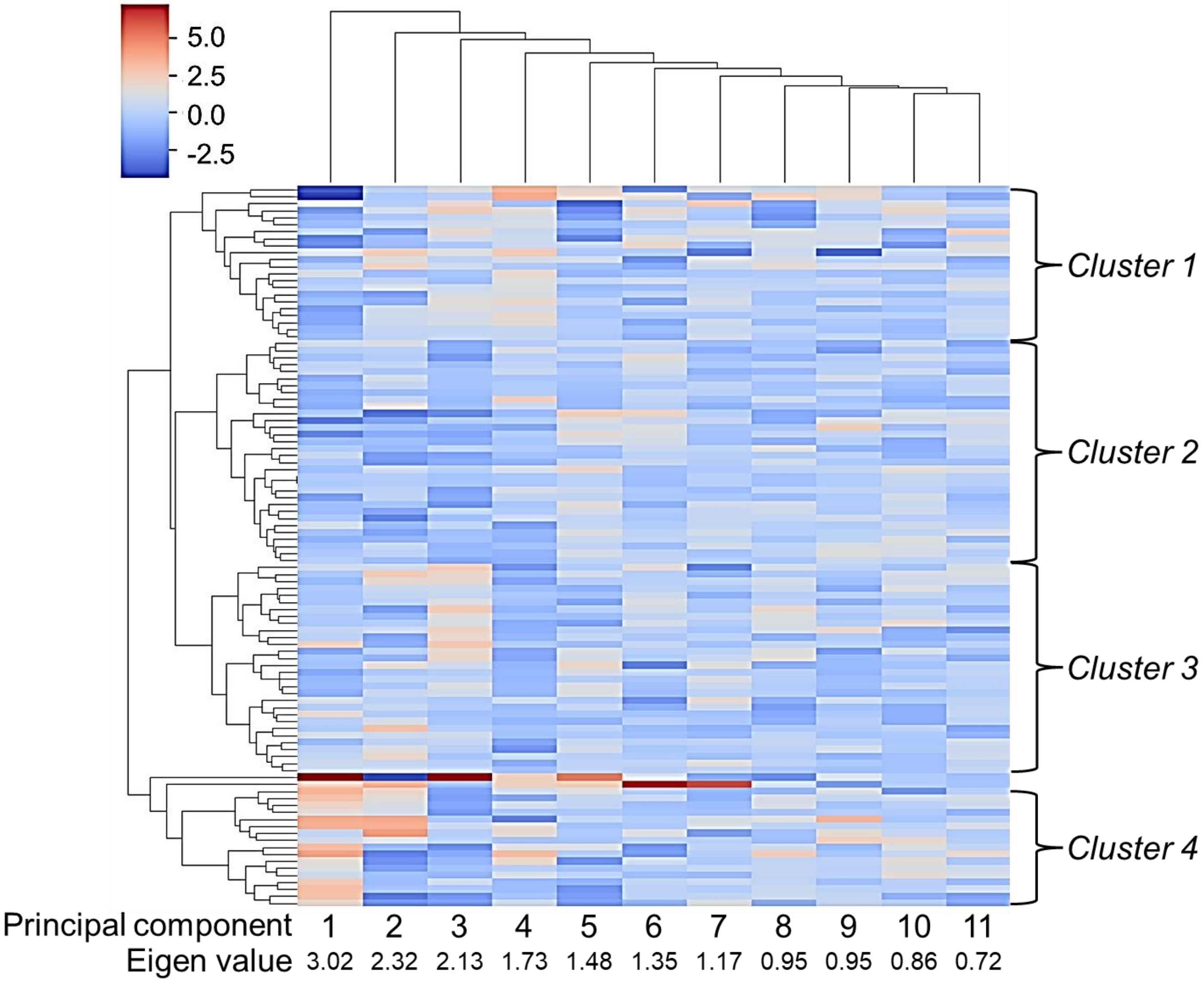

Supplemental Fig. S2 Heatmap in the hierarchical clustering analysis. The horizontal axis indicates 10 principal components for the analysis, and eigen values are shown under each component. The vertical axis indicates each case in each subgroup.
