# Supplementary material for: Heterogeneity in Kawasaki disease patients with coronary artery abnormalities investigated by data-driven cluster analysis
Source: Pediatr Res. 2025 Jun 20;98(5):1809–16. doi: 10.1038/s41390-025-04205-8 (PMC12602351; doi:10.1038/s41390-025-04205-8)
Supplement: Supplementary file 3 — Supplementary Table. S1 [file 41390_2025_4205_MOESM3_ESM.pdf]

Supplemental Table S1. Demographics and distribution of each cluster in 12 facilities

Facilities	No. of KD cases	Male (%)	Age (month) median(IQR)	Days of initial treatment Median(IQR)	No. of cases with CAA in acute phase	Cluster 1 (n=22)	Cluster 2 (n=32)	Cluster 3 (n=30)	Cluster 4 (n=17)
Yamanashi Prefectural Central Hospital	158	54.4	27 (14-42)	5 (5-6)	13 (8%)	1 (8%)	2 (15%)	9 (69%)	1 (8%)
Kofu Municipal Hospital	135	54.8	24 (12-44)	5 (5-6)	24 (18%)	6 (25%)	8 (33%)	6 (25%)	4 (17%)
Yamanashi Kosei Hospital	118	56.8	30 (15-45)	5 (4-6)	8 (7%)	1 (13%)	3 (38%)	2 (25%)	2 (25%)
Yamanashi Red Cross Hospital	59	56.0	21 (14-35)	5 (4-6)	14 (24%)	5 (36%)	4 (29%)	3 (21%)	2 (14%)
National Hospital Organization Kofu National Hospital	53	56.6	30 (14-48)	5 (5-6)	7 (13%)	4 (57%)	1 (14%)	1 (14%)	1 (14%)
Suwa Central Hospital	51	64.7	32 (18-40)	5 (5-5.5)	8 (16%)	1 (13%)	4 (50%)	1 (13%)	2 (25%)
Nirasaki Municipal Hospital	41	51.2	26 (12-41)	5 (4-5)	3 (7%)	1 (33%)	0 (0%)	0 (0%)	2 (67%)
Fujiyoshida Municipal Hospital	38	47.3	30 (17-46)	5 (5-6)	10 (26%)	0 (0%)	4 (40%)	4 (40%)	2 (20%)
Tsuru Municipal General Hospital	27	63.0	20 (13-33)	5 (5-6)	5 (19%)	1 (20%)	3 (60%)	1 (20%)	0 (0%)
Kofu-Kyoritsu Hospital	20	50.0	24 (11-55)	5 (5-6)	5 (25%)	1 (20%)	3 (60%)	1 (20%)	0 (0%)
Kyonan Medical Center Fujikawa Hospital	20	75.0	34 (23-41)	5 (5-5)	2 (10%)	0 (0%)	0 (0%)	1 (50%)	1 (50%)
University of Yamanashi	6	83.3	27 (23-94)	5.5 (5-6)	4 (67%)	1 (25%)	0 (0%)	1 (25%)	0 (0%)

Chi-squared test : $p = 0.23$
