# Supplementary material for: Heterogeneity in Kawasaki disease patients with coronary artery abnormalities investigated by data-driven cluster analysis
Source: Pediatr Res. 2025 Jun 20;98(5):1809–16. doi: 10.1038/s41390-025-04205-8 (PMC12602351; doi:10.1038/s41390-025-04205-8)
Supplement: Supplementary file 4 — Supplementary Table. S2 [file 41390_2025_4205_MOESM4_ESM.pdf]

Supplemental Table S2. List of all 33 continuous variables before starting initial treatment

Categories	Variables
Demographic features	Age, height, weight, BSA,<br>days of initial treatment (start day),<br>the number of major KD symptoms (symptoms)
Laboratory data	White blood cell count (WBC); hemoglobin level (Hb) platelet count (Plt); Serum levels of C-reactive protein (CRP), total protein (TP), albumin (Alb), sodium, potassium, chloride, aspartate aminotransferase (AST), alanine aminotransferase (ALT), lactic acid dehydrogenase (LDH), total bilirubin, blood urea nitrogen (BUN), creatinine (Cre), creatine kinase (CK), globulin (IgG), total cholesterol (T. cholesterol), high density lipoprotein cholesterol (HDL cholesterol), low density lipoprotein cholesterol (LDL cholesterol) non-high density lipoprotein cholesterol (Non HDL cholesterol), and triglyceride (TG); PT-INR, fibrinogen, D-dimer value
Echocardiographic parameters	Maximum coronary artery Z-score before initial treatment (Pre-max CA Z-score), Maximum coronary artery diameter before initial treatment (Pre-max CA diameter)
