# Supplementary material for: Heterogeneity in Kawasaki disease patients with coronary artery abnormalities investigated by data-driven cluster analysis
Source: Pediatr Res. 2025 Jun 20;98(5):1809–16. doi: 10.1038/s41390-025-04205-8 (PMC12602351; doi:10.1038/s41390-025-04205-8)
Supplement: Supplementary file 5 — Supplementary Table. S3 [file 41390_2025_4205_MOESM5_ESM.pdf]

Supplemental Table S3. Number of missing values for each variable used in the cluster analysis

Variables	No. of missing values (%)
Age	0 (0.0)
Days of initial treatment (start day)	0 (0.0)
The number of major KD symptoms	0 (0.0)
White blood cell count (WBC)	0 (0.0)
Platelet count (Plt)	1 (1.0)
C-reactive protein (CRP)	0 (0.0)
Albumin	0 (0.0)
Creatine kinase	0 (0.0)
Aspartate aminotransferase	0 (0.0)
Total bilirubin	5 (4.9)
Blood urea nitrogen	1 (1.0)
Sodium	1 (1.0)
Potassium	1 (1.0)
Total cholesterol	13 (12.6)
High density lipoprotein cholesterol	27 (26.2)
Triglyceride	15 (14.6)
PT-INR	3 (2.9)
Fibrinogen	6 (5.8)
D-dimer	17 (16.5)
Pre-max CA Z-score	0 (0.0)
