# Supplementary material for: Heterogeneity in Kawasaki disease patients with coronary artery abnormalities investigated by data-driven cluster analysis
Source: Pediatr Res. 2025 Jun 20;98(5):1809–16. doi: 10.1038/s41390-025-04205-8 (PMC12602351; doi:10.1038/s41390-025-04205-8)
Supplement: Supplementary file 6 — Supplementary Table. S4 [file 41390_2025_4205_MOESM6_ESM.pdf]

Supplemental Table S4. Baseline demographics, clinical features, and blood laboratory data of patients with coronary artery abnormality (CAA) or without CAA in the acute phase

Demographics, clinical features, and blood laboratory data	CAA(+) in acute phase (n=103)	CAA(-) in acute phase (n=623)	p value*
Age ; months	18 (7-38)	27 (15-44)	< 0.01
< 6months ; n (%)	17 (16.5)	23 (3.7)	< 0.01
< 12months ; n (%)	38 (36.9)	107 (17.1)	< 0.01
Male ; n (%)	55 (53.4)	352 (56.5)	0.12
Days of initial treatment ; day	5 (4-6)	5(4.5-6)	0.80
Incomplete KD ; n (%)	19 (18.4)	56 (9.0)	< 0.01
IVIG resistance ; n (%)	32 (31.1)	119 (19.1)	< 0.01
Resistance of 2nd line treatment ; n (%)	21 (20.4)	22 (3.5)	< 0.01
Plasma exchange ; n (%)	12 (11.7)	7 (1.1)	< 0.01
Pre-max CA diameter ; mm	2.4 (2.0-2.7)	2.0 (1.8-2.3)	< 0.01
Pre-max CA Z-score	2.36 (1.34-2.93)	0.84 (0.27-1.42)	< 0.01
Pre-max CA Z-score > 2.0 ; n (%)	60 (58.3)	6 (1.0)	< 0.01
Pre-max CA Z-score > 2.5 ; n (%)	49 (47.6)	0 (0.0)	< 0.01
Max CA Z-score within 30 days	3.07 (2.69-3.97)	1.19 (0.63-1.75)	<0.01
CAA after 30 days ; n (%)	17 (16.5)	0 (0.0)	< 0.01
White blood cell count (WBC) ; $\times 10^3$ /µL	13.9 (10.5-16.4)	13.0 (10.4-16.1)	0.32
Hemoglobin ; g/dL	11.2 (10.3-11.9)	11.5 (10.9-12.2)	< 0.01
Platelet count ; $\times 10^4$ /µL	34 (27.8-40.5)	32.8 (27.3-39.9)	0.21
Aspartate aminotransferase (AST) ; U/L	34 (27-93)	34 (27-66)	0.62
Alanine aminotransferase (ALT) ; U/L	32 (16-115)	23 (13-94)	0.03
Total bilirubin ; mg/dL	0.6 (0.5-1.0)	0.6 (0.4-0.8)	0.07
Sodium ; mmol/L	134 (132-137)	135 (133-136)	0.40
C-reactive protein (CRP) ; mg/L	9.7 (5.2-13.5)	6.9 (4.0-10.4)	< 0.01
Albumin ; g/dL	3.3 (3.0-3.6)	3.6 (3.3-3.9)	< 0.01
Triglyceride ; mg/dL	94 (77-123)	94 (77-118)	0.49
Total cholesterol ; mg/dL	134 (117-160)	145 (129-162)	< 0.01
HDL cholesterol ; mg/dL	30 (23-37)	33 (26-41)	0.03

Data are expressed as the median (IQR) or n (%). * Kruskal-Wallis or Chi-squared test
